# MutMap+: Genetic Mapping and Mutant Identification without Crossing in Rice

**DOI:** 10.1371/journal.pone.0068529

**Published:** 2013-07-10

**Authors:** Rym Fekih, Hiroki Takagi, Muluneh Tamiru, Akira Abe, Satoshi Natsume, Hiroki Yaegashi, Shailendra Sharma, Shiveta Sharma, Hiroyuki Kanzaki, Hideo Matsumura, Hiromasa Saitoh, Chikako Mitsuoka, Hiroe Utsushi, Aiko Uemura, Eiko Kanzaki, Shunichi Kosugi, Kentaro Yoshida, Liliana Cano, Sophien Kamoun, Ryohei Terauchi

**Affiliations:** 1 Iwate Biotechnology Research Center, Kitakami, Iwate, Japan; 2 United Graduate School of Iwate University, Morioka, Iwate, Japan; 3 The Sainsbury Laboratory, Norwich Research Park, Norwich, United Kingdom; Auburn University, United States of America

## Abstract

Advances in genome sequencing technologies have enabled researchers and breeders to rapidly associate phenotypic variation to genome sequence differences. We recently took advantage of next-generation sequencing technology to develop MutMap, a method that allows rapid identification of causal nucleotide changes of rice mutants by whole genome resequencing of pooled DNA of mutant F2 progeny derived from crosses made between candidate mutants and the parental line. Here we describe MutMap+, a versatile extension of MutMap, that identifies causal mutations by comparing SNP frequencies of bulked DNA of mutant and wild-type progeny of M3 generation derived from selfing of an M2 heterozygous individual. Notably, MutMap+ does not necessitate artificial crossing between mutants and the wild-type parental line. This method is therefore suitable for identifying mutations that cause early development lethality, sterility, or generally hamper crossing. Furthermore, MutMap+ is potentially useful for gene isolation in crops that are recalcitrant to artificial crosses.

## Introduction

Recent developments in next-generation sequencing (NGS) technologies are revolutionizing various aspects of genomics. In particular, NGS-based whole genome analysis of organisms is becoming routine. Draft genome sequences of a large number of organisms including non-model species are rapidly accumulating thanks to the tremendous advances made in de novo sequencing techniques and the development of highly efficient assembly software [1], [2]. Once whole genome draft sequences become available, resequencing of multiple individuals of the same species allows rapid identification of genomic variations, contributing to genetic analyses of medical conditions in human as well as important traits in crops, animals and microbes [3]–[8].

Whole genome sequencing technology promises to dramatically impact crop improvement in this era of looming food crisis and an ever-increasing world population. To exploit genome sequencing in crop breeding, we recently developed a method called MutMap and applied it to the identification of rice genes responsible for agronomically important traits [7]. For MutMap application, we generated over 12,000 mutant lines of M2–M5 generations by ethylmethane sulfonate (EMS) mutagenesis of a Northern Japanese rice (*Oryza sativa* ssp. *japonica*) cultivar “Hitomebore” [9]. The mutant lines show wide variations of phenotypes in agronomically important traits like plant height, grain number and disease tolerance. Using MutMap, we identified the unique genomic positions that harbor mutations causing pale green leaves and semi-dwarfism. Because it relies on crosses to the parental line and eliminates the need for wide-crosses to genetically unrelated lines, MutMap is particularly useful for identifying genes that determine quantitative minor effect phenotypes, which is a major challenge in crop improvement [7].

MutMap is based on the crossing of a mutant of interest to the parental line that was used for the mutagenesis, followed by selfing of F1 individuals to generate F2 progeny. If the phenotype is caused by a single recessive mutation, the F2 population is expected to segregate 3∶1 for the wild-type and mutant progeny. DNA from about 20 F2 individuals showing the mutant phenotype is pooled in an equal ratio and subjected to Illumina whole genome sequencing with depth of more than 10x coverage. The short reads are then aligned to the reference genome sequence constructed for the cultivar used for the mutagenesis, to Hitomebore reference sequence in our case. Since the causal SNP is shared by F2 mutant progeny, all the re-sequenced short reads covering such SNP should have a nucleotide different from the reference sequence. In contrast, SNPs that are not relevant to the phenotype under consideration should segregate in 1∶1 ratio among the F2 progeny. Consequently, about half of the short reads covering such positions contain a nucleotide that is different from the reference genome. To quantify the proportion of short reads having nucleotides different from the reference sequence (SNPs), we developed the concept of SNP-index. If the entire short reads covering a particular genomic position share a SNP that differs from the reference, the SNP-index is defined as 1, whereas if only half of the short reads share such an SNP, then the SNP-index is 0.5. The SNP-index is calculated for all the SNPs incorporated by mutagenesis, and the relationship between SNP-index and genomic position is graphically plotted. The genomic region showing a unique SNP-index peak (SNP-index = 1) corresponds to the position of the causal mutation responsible for phenotype of our candidate mutant.

The MutMap method is based on selecting mutants of interest at M3–M5 generations and crossing them to the parental line, followed by evaluation of phenotypes in segregating F2 progeny. Accordingly, mutants that are not amenable for crossing, *e.g.* mutants with early development lethality or sterility, are not suitable for MutMap application. To address this problem, we developed MutMap+, a versatile extension of MutMap that is based on selfing of heterozygous plants showing wild-type phenotype and identified in M2 progeny segregating for wild-type and a mutant phenotype of interest that is recessive homozygous. The resulting M3 population segregates for the target mutation and can be directly used for whole-genome sequencing. Since MutMap+ circumvents the necessity of backcrossing to the wild-type parent, it is suitable for the isolation of mutations in genes that hamper artificial crossing. Thus MutMap+ expands the applications of MutMap for the genetic improvement of rice and other crop plants.

## Results

### Principle of MutMap+

**Figure 1:**
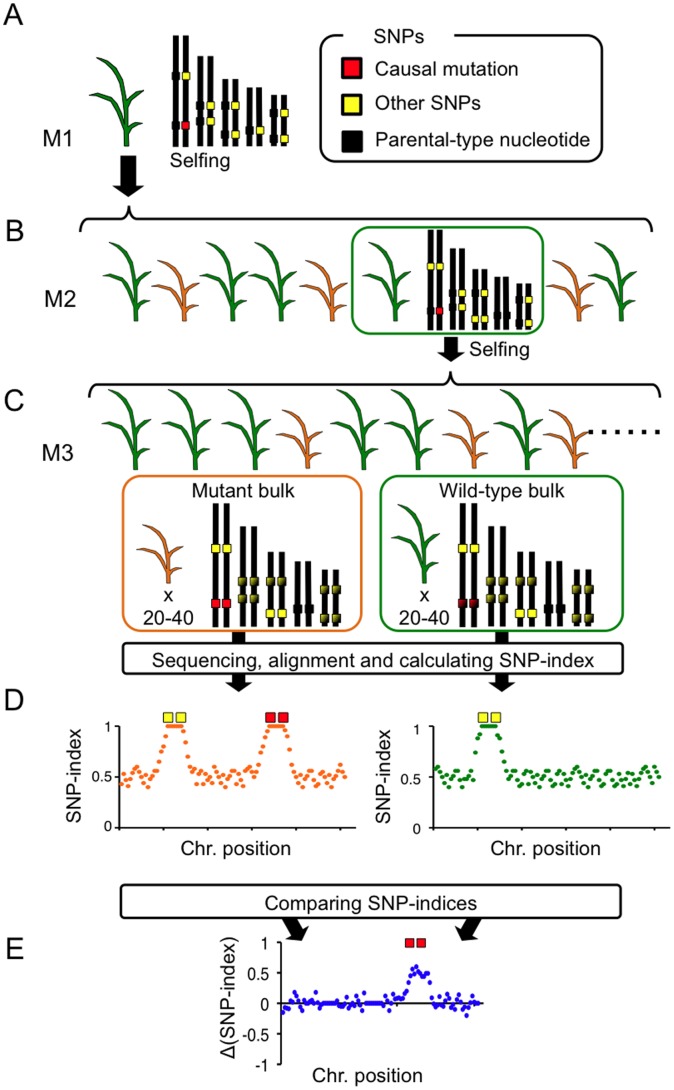
A simplified scheme of MutMap+. (**A**) Seeds harvested following EMS mutagenesis of rice at immature embryo stage are used to establish M1 generation, at which stage most of mutations incorporated by EMS are in the heterozygous state. (**B**) M2 progeny obtained from a self-fertilized M1 plant segregate for wild-type (indicated by green color) and mutant (brown color) phenotypes. Here we focus on wild-type heterozygous individuals. (**C**) Heterozygous M2 plant are selfed to obtain M3 progeny that segregate 3∶1 for wild-type and mutant phenotypes. Genomic DNA from 20–40 M3 mutant and wild-type M3 progeny are separately bulked, and subjected to whole-genome sequencing. The resulting short reads are aligned to reference sequence of the cultivar used for mutagenesis. (**D**) SNP-index is calculated for each SNP, and plots relating SNP-index and chromosome positions are obtained for both the mutant and wild-type M3 bulks separately. The two SNP-index plots are compared to identify the region with SNP-index = 1 that is specific to the mutant bulk. (**E**) We can also evaluate Δ(SNP-index) plot, which is obtained by subtracting SNP-index value of wild-type bulk from that of mutant bulk. Genomic region harboring the causal mutation should have positive Δ(SNP-index) values.

The principle of MutMap+ is explained in **Figure 1** using rice as an example. We applied EMS mutagenesis to rice immature embryo immediately after fertilization, and the embryos were allowed to develop to seeds. These seeds were sown to generate M1 plants, in which the majority of EMS mutations were expected to be in the heterozygous state (**Figure 1A**). Although phenotypes of dominant or semi-dominant mutations could be observed at this generation, M2 seeds obtained from selfing of M1 plants were advanced to M2 generation as our focus was on recessive mutations. We then selected those M2 progeny that segregated 3∶1 for wild-type and mutant phenotypes, respectively (**Figure 1B**). For application of the MutMap+ scheme, we planted ∼10 M2 progeny per line in the field, and scored phenotypic segregation (**Figure 1B**). For the lines segregating for a phenotype of interest, we expected that 2/3 of the wild-type siblings are heterozygous for the causal mutation. These wild-type M2 siblings were carefully grown to generate as many M3 seeds as possible that were harvested separately from individual plants. Over 80 M3 seeds derived from each M2 parent were then sown in the field to observe the segregation of phenotype. M3 families that segregated for the wild-type and mutant phenotypes were selected for further analysis (**Figure 1C**).

Next, we made two bulks of DNA, one from 20–40 M3 individuals showing the mutant phenotype and another from 20–40 M3 individuals with the wild-type phenotype, both of which were derived from selfing of a single heterozygous M2 plant (**Figure 1C**). These two bulks of DNA were separately sequenced by Illumina GAIIx sequencer, and aligned to the reference sequence of the parental cultivar. For each bulk, we generated SNP-index *vs* SNP genomic position graphs (**Figure 1D**).

There are two reasons for the occurrence of genomic regions with SNP-index = 1 in the SNP-index plots obtained for the mutant DNA pool (**Figure 1D**). The first is that the region actually harbors the causal mutation for the phenotype. The second is because SNPs irrelevant to the phenotype become fixed to homozygous state in the M2 generation and thus are shared by all M3 plants. This is expected to happen in 50% of the total genomic region. It is not possible to discriminate between the two types of SNPs by simply looking at the SNP-index plot of the mutant bulk. However, it is possible to identify those SNPs with SNP-index = 1 that are randomly fixed to homozygous state (second case) by comparing the SNP-index plots of the wild-type and mutant bulks. Regions showing SNP-index = 1 by random fixation of SNPs in the M2 generation should be shared between the two bulks, whereas the region harboring the causal mutation should be specific to the mutant bulk. Regions with SNP-index of 1 resulting from the two types of SNPs should be mutually exclusive since the M2 individual used for generating the M3 progeny sequenced is heterozygous for the region harboring the causal mutation. In practice, we scan the SNP-index plots of the mutant and wild-type bulks to find regions with SNP-index = 1 specific to the mutant bulk (**Figure 1D**). To visualize this data, we subtract the SNP-index of the wild-type bulk from that of mutant bulk for each SNP to obtain a Δ(SNP-index) (**Figure 1E**). Δ(SNP-index) value should be around 0 for most of the genome, but it is significantly positive in the region harboring the causal mutation. To assess the statistical significance of the Δ(SNP-index) values, we apply Fisher’s exact test [10].

### Application of MutMap+ for the Identification of Causal SNPs in Rice Mutants

**Figure 2:**
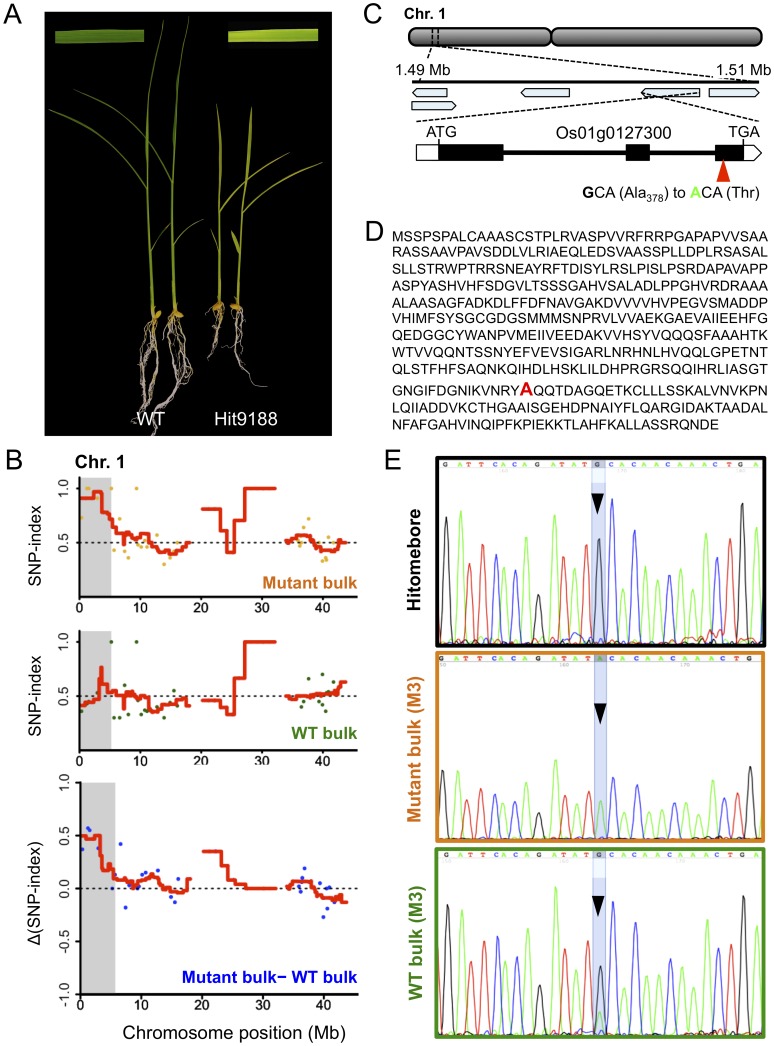
MutMap+ identifies the causal mutation of the Hit9188 early stage lethality phenotype. (**A**) The Hit9188 mutant is characterized by seedlings with pale-green and dwarf phenotypes that eventually die out starting from about three weeks after germination. (**B**) Chromosome 1 SNP-index plot for mutant and wild-type (WT) bulks derived from a segregating Hit9188 M3 progeny that was obtained by selfing a heterozygous M2 plant, as well as the Δ(SNP-index) plot generated by subtraction of WT bulk from mutant bulk. Points in the graphs represent SNPs, and the red lines represent the sliding window average of 4 Mb interval with 10 Kb increment. Shaded areas correspond to the genomic region where SNP-indices of mutant bulk and wild-type bulk show statistically significant (*P*<0.05) differences (*i.e.* Δ(SNP-index) >0). (**C**) Genomic location and structure of the Os01g0127300 gene harboring the Hit9188 mutant candidate nucleotide change, whose position within the gene together with the predicted amino acid change are indicated by a red triangle. (**D**) The deduced amino acid sequence of the protein encoded by Os01g0127300. The red font indicates the mutated alanine residue. (**E**) DNA sequencing peak chromatograms of the region spanning the Os01g0127300 mutation showing the wild-type G in Hitomebore, mutant A in mutant bulk, and the heterozygous G/A in wild-type (WT) bulk.

As a proof of concept, we applied MutMap+ to a rice mutant Hit9188 derived from EMS mutagenesis of cultivar Hitomebore. This mutant, identified among ten M2 plants that originated from a self-fertilized M1 individual planted in the paddy field, is characterized by dwarfism and pale green leaves followed by premature death 3 weeks after germination (**Figures 2A**, **Figure S1**). Thus, the mutant could not be crossed to the parental line Hitomebore to apply the classical MutMap analysis. As a result, wild-type M2 siblings of the mutant were grown to maturity and allowed to self-fertilize. We obtained over 100 seeds/plant, which were sown separately to generate M3 progeny that were further assessed for phenotypic segregation. One M3 progeny segregated 167 (wild-type) to 56 (mutant-type), conforming to the 3∶1 ratio (Chi square test: χ^2^ = 0.0015, ns). This indicated that the mutant phenotype is caused by a single recessive mutation. We then made two bulks of DNA; a mutant bulk of 40 mutant-type progeny and a wild-type bulk of 40 wild-type progeny. The mutant bulk and the wild-type bulk were separately sequenced to generate Illumina GAIIx 75bp paired end reads. We obtained 95,552,424 and 57,925,150 short reads corresponding to 7.17 Gbp and 4.34 Gbp sequence reads for the mutant and wild-type bulks, respectively (**Table S1**).

The Illumina short reads obtained for the two DNA bulks (DDBJ Accession Read Archive: DRA001007) were separately aligned to the reference sequence of Hitomebore (DDBJ Accession Read Archive: DRA000927) and the SNP-index was calculated for each SNP [7]. The relationship between the SNP-index and chromosome position was plotted for the 12 rice chromosomes as shown in **Figures S2A and B**. To help visualize the patterns of SNP-index, we applied a sliding-window analysis by taking the average value of SNP-index over 4 Mb window and incrementing the window 10 Kb at a time. The SNP-index plots were very similar between the mutant and wild-type bulks across the entire genome (**Figures S2A and B**). However, we identified a single unique region on chromosome 1 (between 0 and 5.23 Mb) with a peak of SNP-index close to 1 in the mutant bulk that is missing from the wild-type bulk (**Figure 2B**, **Figures S2A and B**), and this could be readily visualized using a Δ(SNP-index) graph (**Figure S2C**). As expected, Δ(SNP-index) was close to 0 across the genome, but within the unique genomic region identified on chromosome 1,0–5.23 Mb, its value was greater than zero. This was the only region that exhibited a SNP-index difference of >0 that is statistically significant between the mutant and wild-type bulks (Fisher’s exact test: *P*<0.05).

After identifying the region with SNP-index = 1 that is specific to the mutant bulk, we scrutinized in detail the SNPs therein (Chromosome 1,0–5.23 Mb). Accordingly, we found a total of 8 SNPs with SNP index = 1 in the region for the mutant bulk (**Table S2**). Of these, only two SNPs (nucleotide 1234738 and 1503571) were nonsynonymous causing amino acid changes (**Table S2**). The first mutation (nucleotide 1234738), a C to T transition, causes amino acid change from proline to leucine in Os01g0121800, a gene encoding a glycosyl transferase family 14 protein (**Table S2**). The second SNP causes amino acid changes in a 482 amino acid protein encoded by Os01g0127300 (**Figure** **2C**). This SNP (C in Hitomebore to T in Hit9188: G to A in sense-strand) alters amino acid 378 from alanine to threonine (**Figure** **2D**). Because of the type of proteins encoded by the two genes harboring the mutations detected in Hit9188 and the phenotype of the mutant, we suspected that the second mutation is the most likely candidate, and thus focused on this SNP for further analysis. We used amplicon sequencing with the Sanger method to confirm that this SNP at position 1503571 is G in Hitomebore wild-type, A in the mutant bulk, and a mixture of G and A in the wild-type bulk (**Figure** **2E**).

**Figure 3:**
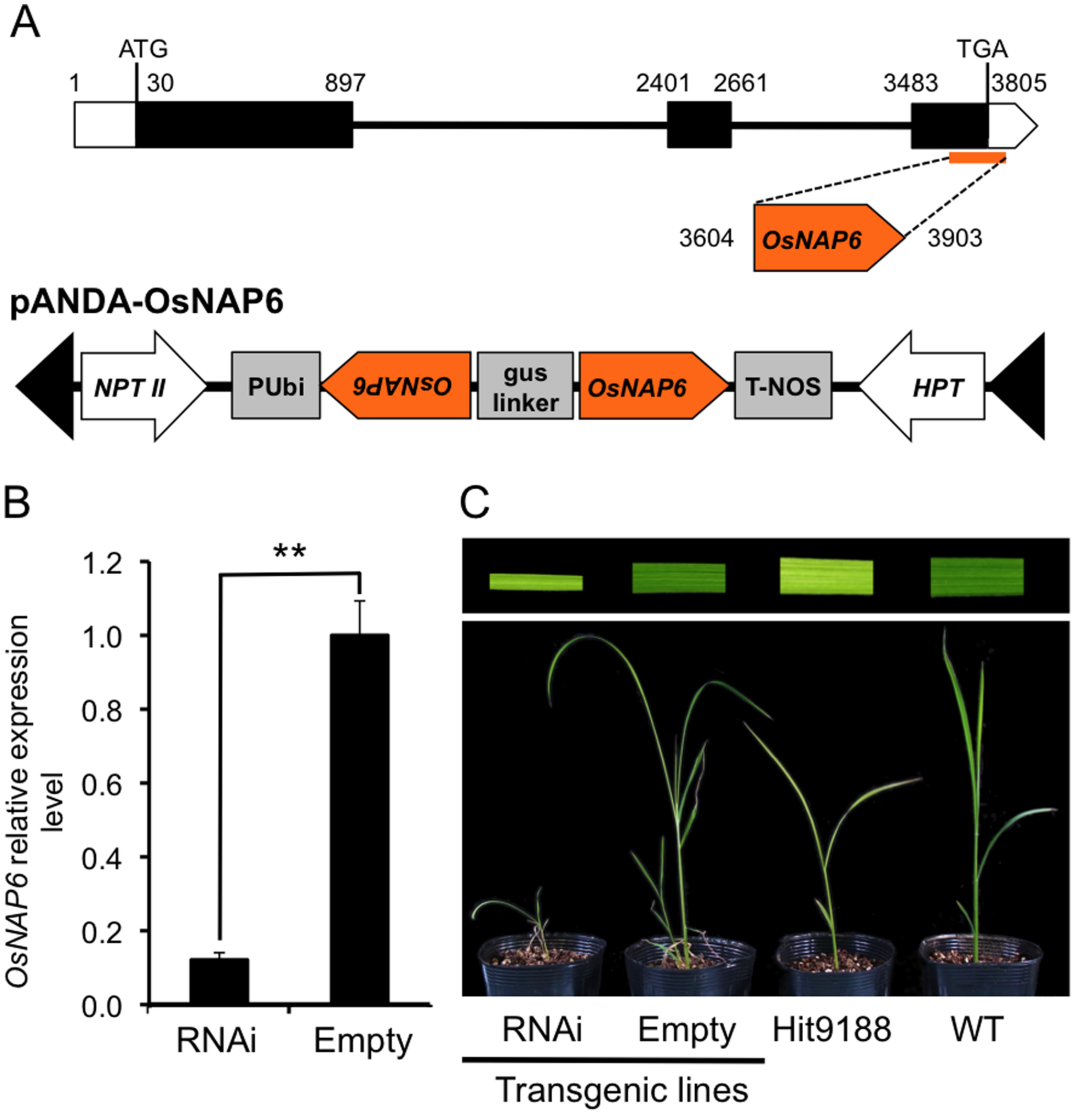
RNA interference confirms that the Os01g0127300 mutation is responsible for Hit9188 developmental phenotypes. (**A**) Structure of Os01g0127300 (*OsNAP6*) and scheme of the construct used for RNAi analysis targeting the *OsNAP6* gene. (**B**) Results of real-time quantitative reverse transcription (RT)-PCR showing the relative expression level of *OsNAP6* in rice plants transformed with OsNAP6-RNAi construct (RNAi) and empty vector (Empty). *Asterisks* indicate significant differences (Student’s *t*-test, **P<0.01). (**C**) Phenotype of leaf blade (top) and seedlings (bottom) of *OsNAP6* RNAi transgenic plants compared to the Hit9188 mutant and Hitomebore wild-type (WT) plants.

The Os01g0127300 gene is predicted to encode a member of the SufBD protein and is homologous to the *Arabidopsis thaliana NAP6* (*Non-intrinsic ABC protein 6*) gene encoding a SufD protein [11]. We thus named the gene and the protein as *OsNAP6* and OsNAP6, respectively. To determine whether the mutation detected in *OsNAP6* is responsible for the Hit9188 mutant phenotypes, we used RNA interference to knockdown the expression of *OsNAP6*. Accordingly, we cloned a 300-bp sequence spanning the last exon and 3′-UTR of *OsNAP6* into pANDA vector [12] generating pANDA-OsNAP6 (**Figure** **3A**). Callus of a rice cultivar Hitomebore was transformed with pANDA-OsNAP6 by *Agrobacterium tumefaciens*. The rice transgenic lines (RNAi-OsNAP6) with reduced expression of *OsNAP6* as determined by quantitative RT-PCR (**Figure** **3B**) exhibited extreme dwarfism, pale green leaves and early death, which is similar to phenotypes of the Hit9188 mutant (**Figure** **3C**). From this result, we concluded that the mutation detected in *OsNAP6* is responsible for the Hit9188 developmental phenotypes.

OsNAP6 shares considerable similarity with proteins from multiple plant species and the mutated alanine residue, which is located within the predicted putative β-helix domain, is conserved (**Figure S3**) [11]. The *A. thaliana nap6* T-DNA insertion mutant shows various developmental phenotypes such as reduced chlorophyll content, shorter roots, a considerable proportion of abnormal embryos, and altered thylakoids [11]. Seedlings with reduced chlorophyll content that eventually die out from about three weeks after sowing also characterize the Hit9188 mutant reported here (Figure S1). The SUF (mobilization of sulfur) system, which contains six proteins (SufA, SufB, SufC, SufD, SufS and SufE), is one of the three systems involved in the biosynthesis, assembly, maturation and repair of iron-sulfur (Fe-S) cluster that is involved in numerous important biological processes [13]−[15].

**Figure 4:**
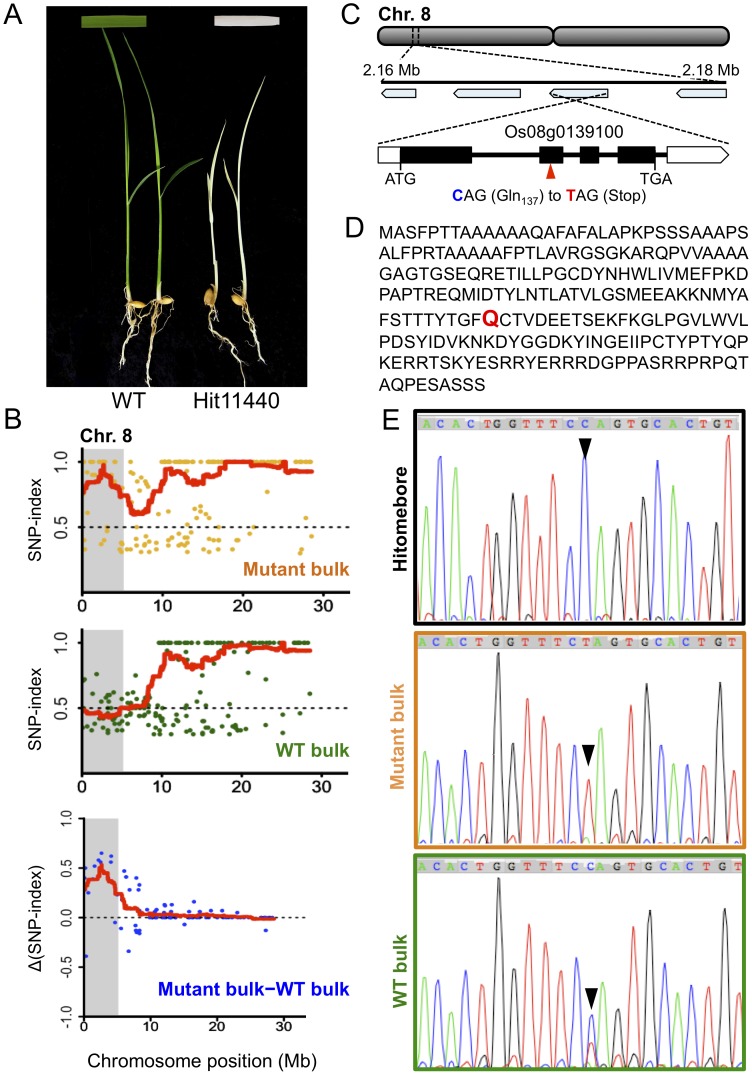
MutMap+ identifies the genomic region harboring the Hit11440 mutation. (**A**) Phenotype of wild-type (WT) and Hit11440 mutant seedlings at about 12 days after sowing. (**B**) Chromosome 8 SNP-index plots for mutant and wild-type (WT) bulks derived from DNA bulks of M3 progeny obtained from selfing of a heterozygous M2 plant, and the Δ(SNP-index) plot generated by subtracting the WT bulk SNP-indices from that of the mutant bulk. Points in the graphs represent SNPs, and red lines represent the sliding window average of 4 Mb interval with 10 Kb increment. Shaded areas correspond to the genomic region where SNP-indices of WT and mutant bulks show statistically significant (P<0.05) differences (i.e. Δ(SNP-index) >0). (**C**) Genomic location and structure of the candidate gene, Os08g0139100, harboring a nucleotide change in the Hit11440 mutant. Location of the mutated glutamine residue is indicted by a red triangle. (**D**) The predicted 299-amino acid sequence of the DAG protein encoded by Os08g0139100. The mutated glutamine (Q) residue is indicated in red. (**E**) Sanger sequencing confirms the candidate SNP identified by Illumina whole genome re-sequencing as indicated by peak chromatograms of the region spanning the Os08g0139100 mutation. The wild-type C in Hitomebore, the mutated T in mutant-bulk DNA, and the C/T mixture in wild-type (WT) bulk DNA are indicted by the black arrows.

We further applied MutMap+ to a second mutant Hit11440 that is characterized by albino seedlings that eventually die from about two weeks after germination (**Figure 4A**). M3 seeds harvested from a heterozygous M2 individual identified among 10 M2 plants were used to establish 104 M3 progeny that segregated 81 (wild-type) to 23 (mutant-type), confirming to 3∶1 ratio (Chi square test: χ^2^ = 0.462, ns). For MutMap+ application, we prepared two DNA bulks; the first bulk composed of 20 M3 mutant albino progeny and the second bulk composed of 20 wild-type M3 progeny mixed in equal proportion and sequenced to a depth of 12 and 11.4 fold coverage, respectively (**Table S1**). SNP-indices were calculated for both bulks and plotted for all the 12 rice chromosomes. Comparison of SNP-index plots of the two bulks as well as generation and further evaluation of Δ(SNP-index) plots identified a 4.34 Mb region (0.32 Mb - 4.66 Mb) on chromosome 8 that is unique to the mutant bulk (**Figure 4B**, **Figure S4**).

We assessed the SNPs within the candidate region on chromosome 8 in detail and identified 10 SNPs with SNP index of 1 **(Table S3**). Of these, only a single SNP causing a G (Hitomebore) to A (Hit11440) transition, which corresponded to a C to T substitution due to opposite orientation of the gene, at the 2,178,176^th^ nucleotide position was non-synonymous. The SNP introduced a premature stop codon at the 137^th^ residue (CAG-Gln to TAG-Stop) of Os08g0139100, a gene predicted to encode a protein similar to the chloroplast precursor Differentiation And Greening (DAG) protein (**Figure 4C**). The protein contains a predicted 27-aa chloroplast transit peptide at its N-terminus (http://www.cbs.dtu.dk/services/SignalP/) and shares considerable homology with DAG proteins from multiple plant species (**Figure S5**). The mutated glutamine residue is also highly conserved among the species considered. The *DAG* locus was previously isolated in *Antrrhinum majus* by transposon tagging, and the corresponding *dag* mutant is characterized by a slow growth and variegated leaves [16], [17], and is among the many examples of variegation mutants caused by transposable element activity [18]. The expression of *DAG* is reported to be essential for chloroplast development, and is required for the expression of the nuclear encoded light harvesting chlorophyll a/b-binding (*CAB*) and *RBCS* genes, as well as for the expression of the plastid gene *RPBO* [16]. Taken together, our finding and the already known function of DAG protein strongly suggest that the mutation identified in Os08g0139100 is the most likely candidate responsible for the albino and early death phenotypes of Hit11440.

## Discussion

We recently reported a whole genome sequencing (WGS)-based method called MutMap that accelerates the identification of genomic regions harboring a causal mutation for a given phenotype in rice. The method can generally be extended to other crop plants provided that the prerequisite of one backcross to the parental line is met. Here we describe a variant of MutMap called MutMap+, which we applied to rice mutants to rapidly identify the causal mutations by whole genome resequencing and comparison of SNP-index plots of two bulked DNA obtained from a segregating M3 progeny. Notably, these experiments did not involve crossing between the candidate mutants and wild-type plants, therefore providing new applications that were not possible with the original MutMap protocol.

The fact that no crossing is required is a significant advantage of MutMap+. Genetic analysis of early stage lethality or sterility genes has remained time consuming. For instance, heterozygous sibling of a mutant of interest is usually crossed to a wild-type plant of a distantly related cultivar to obtain F1, which is selfed to generate F2. If the F2 segregates for the phenotype under investigation, one could map the mutation with DNA markers. In this traditional scheme, at least a cross and selfing followed by linkage analysis with DNA markers is needed. The recently reported method of SNP-Ratio Mapping (SRM) [19] allows rapid identification of lethal alleles with the help of next generation sequencing, but the method still requires crossing. MutMap+ circumvents the need for both crossing and linkage analysis, and therefore can be implemented over a shorter time span. We foresee that this technique can systematically be applied to rapidly identify the genes involved in the control of early plant development and fertility.

MutMap+ is conceptually similar to QTL-seq [20], a WGS-based method we recently reported to rapidly identify quantitative trait loci (QTL) using progeny derived from crosses made between genetically different cultivars. Phenotypic values of quantitative traits among the progeny derived from inter-cultivar crosses usually follow the normal (Gaussian) distribution. In QTL-seq, we make two bulks of DNA, one (H-bulk) from the progeny showing higher values and the other (L-bulk) from the progeny with lower values for the phenotype under consideration. DNA of H- and L-bulks are separately applied to WGS, the resulting short reads are aligned to the reference sequence of either of the parents, and SNP-index plots of H-bulk and L-bulk are compared. Genomic regions displaying contrasting patterns of SNP-index plots between the two bulks indicate the positions of QTLs. Although experimental procedures of MutMap+ and QTL-seq both involve comparison of SNP-index plots of two DNA bulks, MutMap+ addresses a single mutation induced in the genome that results in a discrete phenotypic change. On the other hand, QTL-seq focuses on multiple genetic changes that have accumulated between two cultivars over a much longer time span. Accordingly, the goals of the two methods are different; MutMap+ is used for identification of a single causative nucleotide change responsible for the phenotype, whereas QTL-seq is employed for mapping of approximate positions of multiple QTL.

The application of MutMap+ is not restricted to genes associated with lethality and sterility as described above. This technique could also be widely used to identify genes involved in all types of agronomically important traits. The prerequisite is the availability of draft genome sequences to generate accurate SNP-index plots. The fact that MutMap+ allows gene isolation without crossing has an additional important practical implication. There are many crop species for which efficient techniques of artificial crossing have not been established. For instance, in many millet species including foxtail millet (*Setaria italica*), fonio (*Digitaria exilis*) and tef (*Eragrostis tef*), it is not easy to carry out crosses due to small flower sizes and difficulty in emasculation. MutMap+ can be easily applied to these species that are important to agriculture in developing countries. In view of the projected progress in de novo sequencing technologies and the ever-decreasing sequencing costs, we believe that MutMap+ is poised to be applicable for the improvement orphan crops. The improvement of neglected crops species and varieties should contribute to world food security by enhancing locally adapted agricultural systems.

One of the key advantages of the MutMap approach is the ability to rapidly identify mutations affecting quantitative traits in crop genomes, a limiting feature in many breeding programs. This advantage is conferred by the fact that the crosses are made to the wild-type plant with only the EMS-derived SNPs (on average 1,500 per genome in our rice mutant population) used in genetic mapping. MutMap+ offers the same advantage as the classical MutMap protocol. Because it is based on selfing, it enables precise and robust phenotyping of minor effect traits.

## Materials and Methods

### Plant Materials

The rice (*Oryza sativa* L.) mutants used in this study, Hit9188 and Hit11440, were identified in M2 mutant population generated by ethyl-methanesulfonate (EMS) treatment of immature embryo of an elite *japonica* cultivar Hitomebore. Details of the mutagenesis protocol are provided elsewhere [9]. For identification of candidate mutants for MutMap+ application, about 10 individuals from each M2 line were germinated and grown in the paddy field. Of these, the lines that segregated for mutant and wild-type phenotypes were selected. Growing about 100 seeds from each of the M2 mutant plants showing wild-type phenotypes allowed identification of heterozygous M2 individual plants, which were further used for establishing segregating M3 progeny utilized for making the wild-type and mutant bulks for Illumina sequencing.

### Whole Genome Sequence of Bulked DNA

Genomic DNA extracted from 100 mg of fresh leaf sample of each M3 individual selected using the DNeasy Plant Mini Kit (QIAGEN Sciences) was mixed in an equal ratio to make the bulk-DNA used for sequencing. The library for Illumina sequencing was constructed from five micrograms of DNA sample and sequenced for 76 cycles on an Illumina Genome Analyzer IIx as described in Abe et al. [7]. The sequences are available from DDBJ Sequence Read Archive: DRA001007.

### Alignment of Short Reads to Reference Sequence and SNP Calling

We aligned the short reads from bulked DNA to the reference genome of the cultivar Hitomebore (DDBJ Sequence Read Archive: DRA000927) using BWA (Burrows-Wheeler Aligner) software [21]. Alignment files were converted to SAM/BAM files using SAMtools [22], and applied to the SNP-calling filter “Coval” we previously developed (Kosugi et al. unpublished data; Abe et al. [7]) to increase SNP-calling accuracy. Following SNP-index calculation for all SNP positions, we excluded SNPs with SNP-index of <0.3 from the analysis as they may represent spurious SNPs called due to sequencing and/or alignment errors. For calculating Δ(SNP-index), we only used the SNPs detected in both bulked DNAs.

### Sliding Window Analysis

Sliding widow analysis was applied with 4 Mb window size and 10 Kb increment using an R script that we developed for this purpose. In this sliding window analysis, we calculated average SNP-index and average *P*-value in Fisher’s exact test for the SNPs located in the window. Finally we mapped the causal mutation in the window exhibiting the average *P*-value <0.05.

### RNAi Experiments

For RNAi analysis targeting the *OsNAP6* gene, a 300 bp *OsNAP6* partial fragment was amplified from wild-type Hitomebore cDNA by PCR and subcloned into the binary vector pANDA [12] to yield pANDA-NAP6 (**Figure 3A**). Binary vectors were introduced into Agrobacterium strain EHA105, which as used to transform Hitomebore plants as previously described [23].

## Supporting Information

Figure S1 **The early senescence and premature death phenotype of Hit9188.** Phenotype of Hiotmebore wild-type (left) and Hit9188 (right) plants (**A**) 14 DAS (days after sowing), (**B**) 18 DAS and (**C**) 22 DAS. Bar = 10 cm.
(PPTX)

Figure S2 **MutMap+ identifies the genomic region harboring the causative mutation of Hit9188 (A) SNP-index plot of mutant bulk, (B) SNP-index plot of wild-type bulk, and (C) Δ(SNP-index) plot obtained by subtraction of wild-type bulk SNP-index from mutant bulk SNP-index for all the 12 rice chromosomes.** Red lines represent the sliding window average of 4 Mb interval with 10 Kb increment. Shaded areas correspond to the candidate genomic region where mutant and wild-type SNP-indices exhibit statistically significant (*P* < 0.05) differences (*i.e.* Δ(SNP-index) > 0).
(PPTX)

Figure S3 **Alignment of NAP6 homologs from multiple plant species.** Identical and similar amino acids are indicated in black and gray backgrounds, respectively. Position of the mutated alanine residue in Hit9188 is given by a red box.
(PPTX)

Figure S4 **MutMap+ identifies the genomic region harboring the causative mutation of Hit11440.** (**A**) SNP-index plot of mutant bulk, (**B**) SNP-index plot of wild-type bulk, and (**C**) Δ(SNP-index) plot obtained by subtraction of wild-type bulk SNP-index from mutant bulk SNP-index for all the 12 rice chromosomes. Red lines represent the sliding window average of 4 Mb interval with 10 Kb increment. Shaded areas correspond to the candidate genomic region where mutant and wild-type SNP-indices exhibit statistically significant (*P* < 0.05) differences (*i.e.* Δ(SNP-index) > 0).
(PPTX)

Figure S5 **Alignment of DAG proteins from multiple plant species.** The predicted 27-aa chloroplast residue in rice is underlined, and the highly conserved mutated glutamine (Q) residue in Hit11440 is indicated by a red box. NCBI reference numbers of the sequences used: *Oryza sativa* (NP_001060965.1), *Brachypodium distachyon* (XP_003573360.1), *Hordeum vulgare* (BAJ99034.1), *Sorghum bicolor* (XP_002445021.1), *Zea mays* (ACN27869.1), *Antirrhinum majus* (Q38732.1), *Arabidopsis thaliana* (AAM65001.1), *Glycine max* (ACU13265.1), *Lotus japonicus* (AFK45843.1), *Medicago truncatula* (XP_003593397.1), *Populus trichocarpa* (XP_002316698.1), *Ricinus communis* (XP_002518590.1), and *Vitis vinifera* (XP_002283211.1).
(PPTX)

Table S1 **Summary of Illumina GAIIx whole genome sequencing results of Hit9188 and Hit11440 M3 mutant and wild-type bulks.**
(DOCX)

Table S2 **SNPs with SNP-index 1 within the candidate genomic region detected on chromosome 1 that exhibited statistically significant (Fisher’s exact test:** ***P*** **<0.05) differences between Hit9188 mutant and wild-type bulk sequences.**
(DOCX)

Table S3 **SNPs with SNP-index 1 within the candidate genomic region detected on chromosome 8 that exhibited statistically significant (Fisher’s exact test:** ***P*** **<0.05) differences between Hit11440 mutant- and wild-type bulk sequences.**
(DOCX)
