# Supplementary figures and images for: MutMap+: Genetic Mapping and Mutant Identification without Crossing in Rice
Source: PLoS One. 2013 Jul 10;8(7):e68529. doi: 10.1371/journal.pone.0068529 (PMC3707850; doi:10.1371/journal.pone.0068529)

## Slide 1
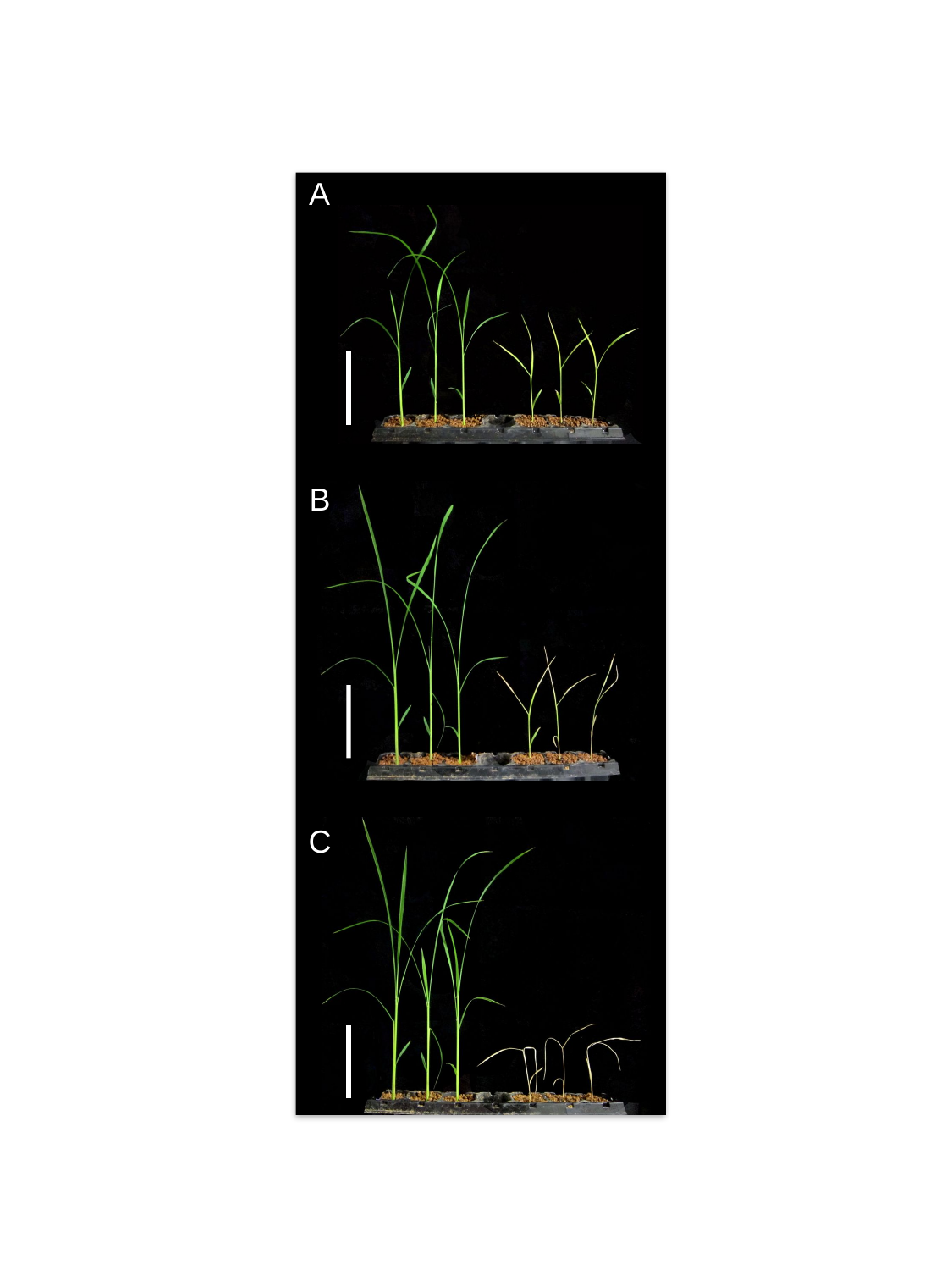

A
B
C

Supplement: Figure S1 — The early senescence and premature death phenotype of Hit9188. Phenotype of Hiotmebore wild-type (left) and Hit9188 (right) plants (A) 14 DAS (days after sowing), (B) 18 DAS and (C) 22 DAS. Bar = 10 cm. (PPTX) [file pone.0068529.s001.pptx]

## Slide 1
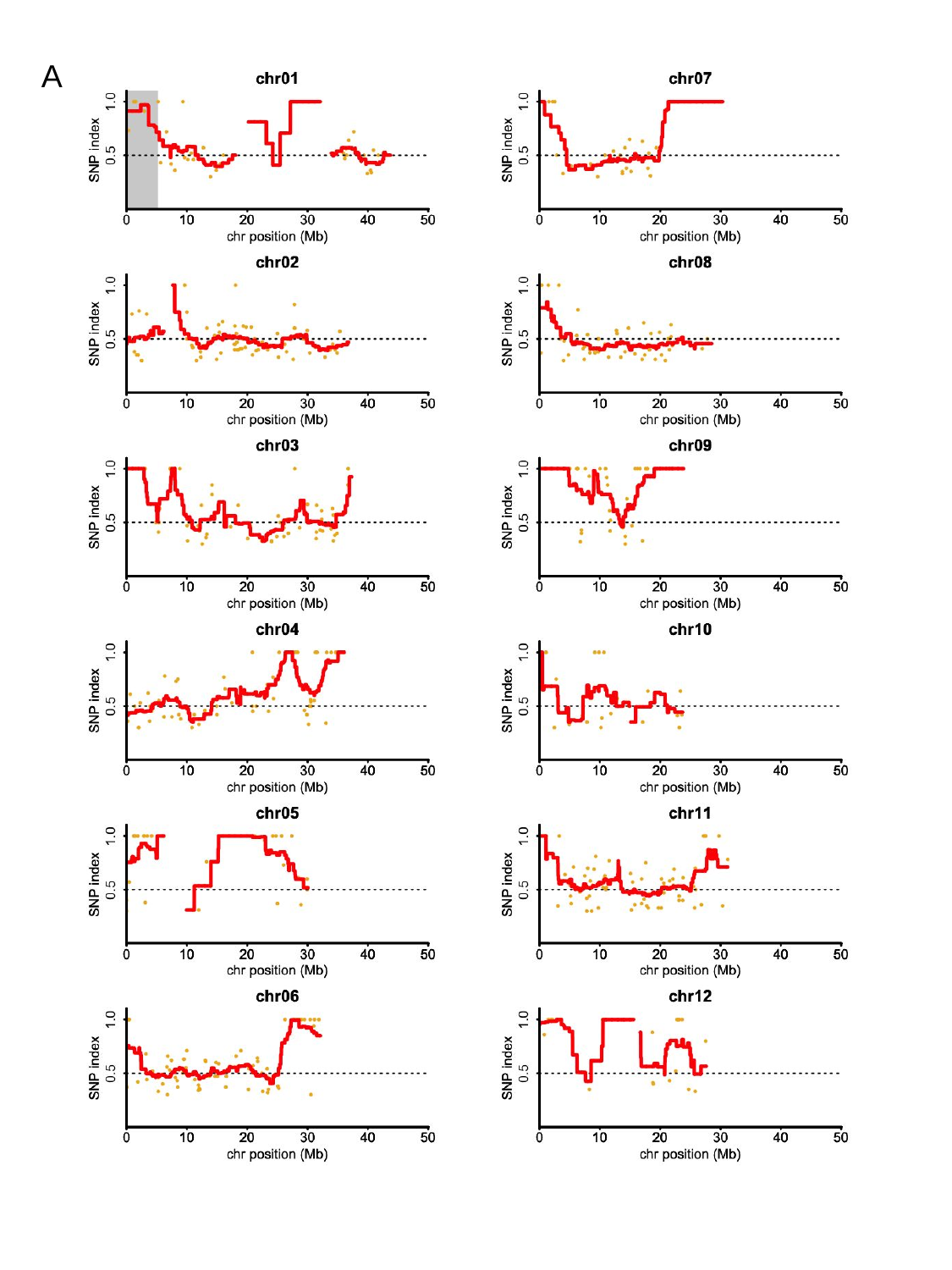

A

## Slide 2
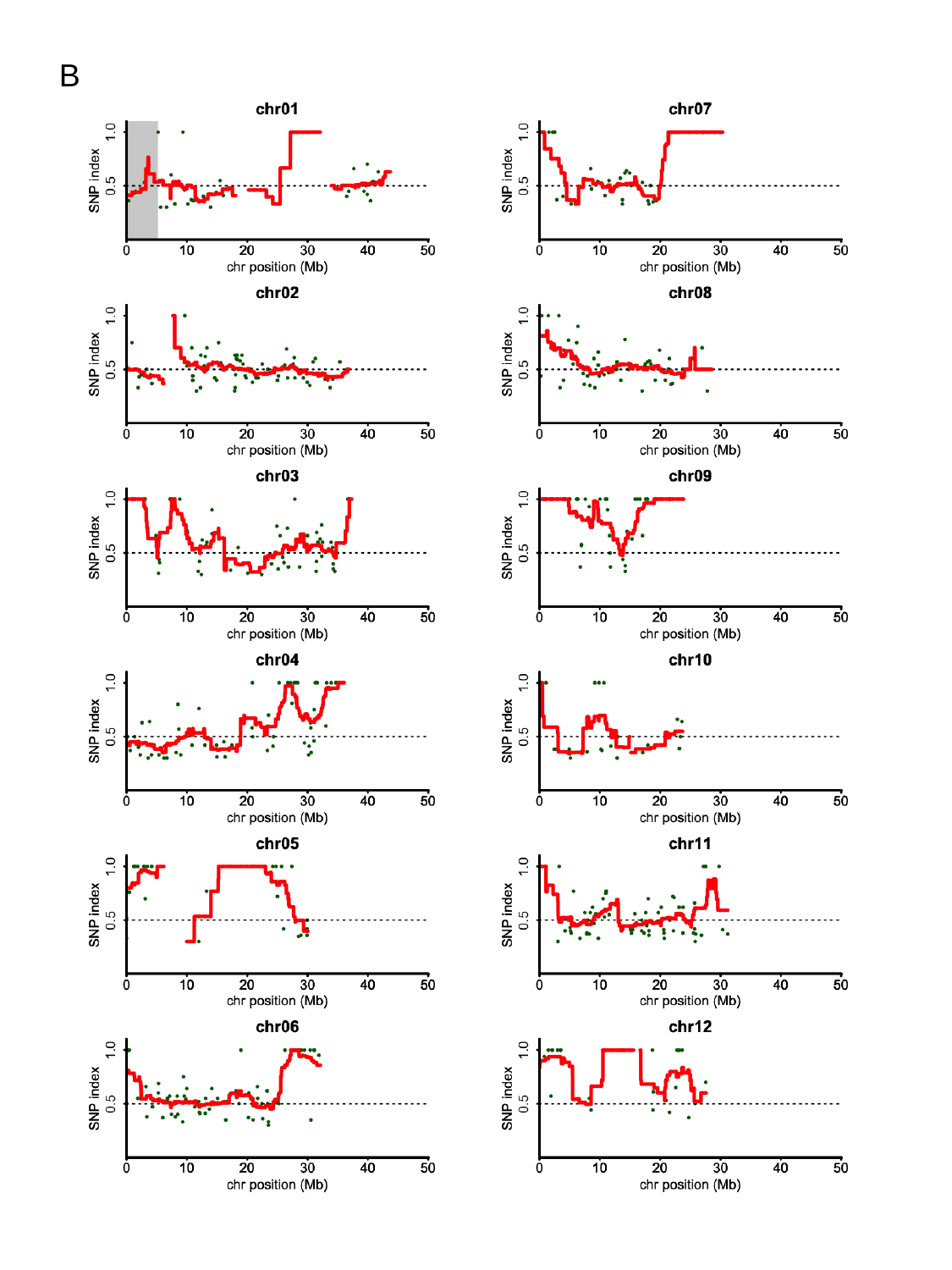

B

## Slide 3
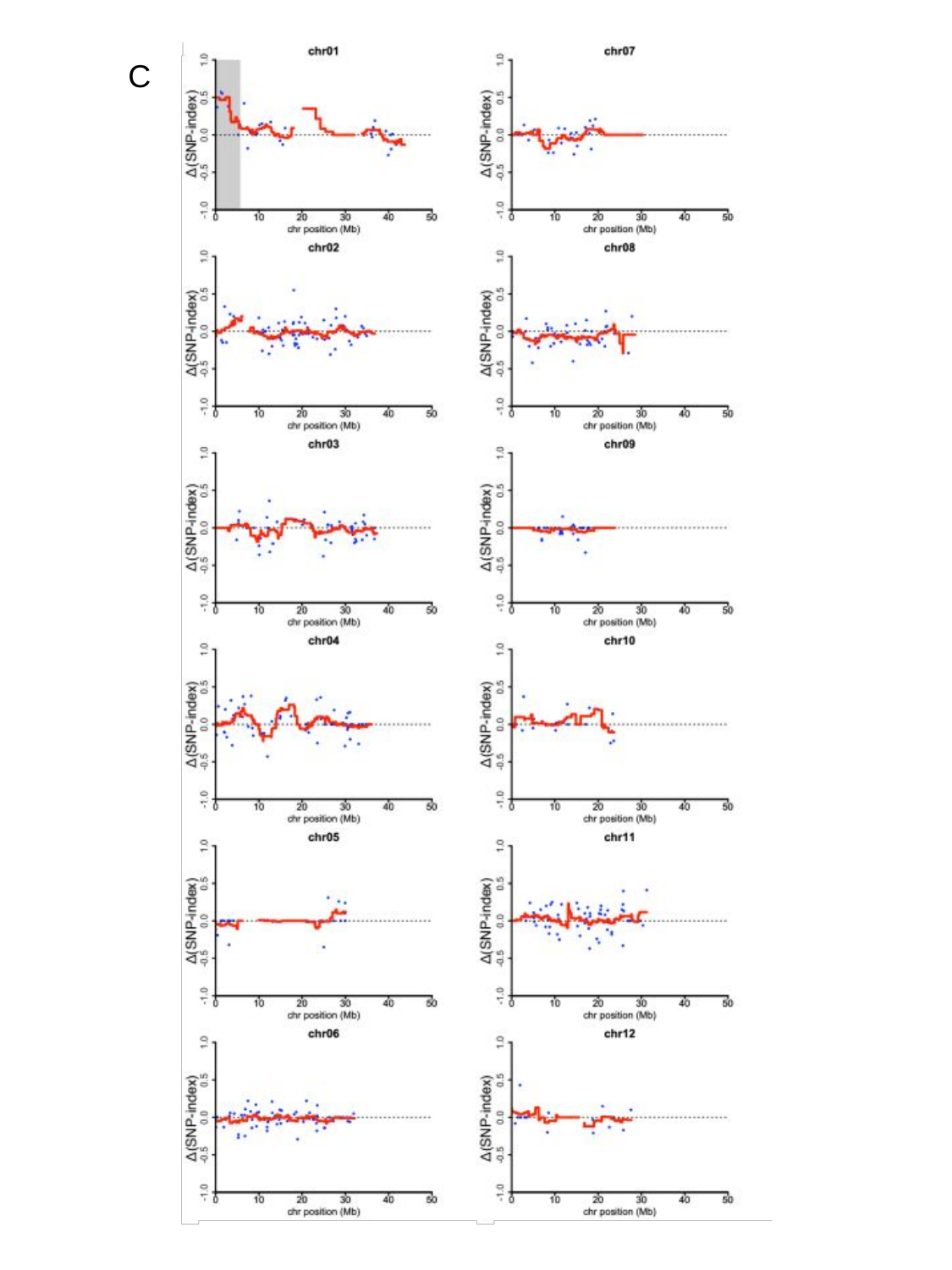

C

Supplement: Figure S2 — MutMap+ identifies the genomic region harboring the causative mutation of Hit9188 (A) SNP-index plot of mutant bulk, (B) SNP-index plot of wild-type bulk, and (C) Δ(SNP-index) plot obtained by subtraction of wild-type bulk SNP-index from mutant bulk SNP-index for all the 12 rice chromosomes. Red lines represent the sliding window average of 4 Mb interval with 10 Kb increment. Shaded areas correspond to the candidate genomic region where mutant and wild-type SNP-indices exhibit statistically significant (P < 0.05) differences (i.e. Δ(SNP-index) > 0). (PPTX) [file pone.0068529.s002.pptx]

## Slide 1
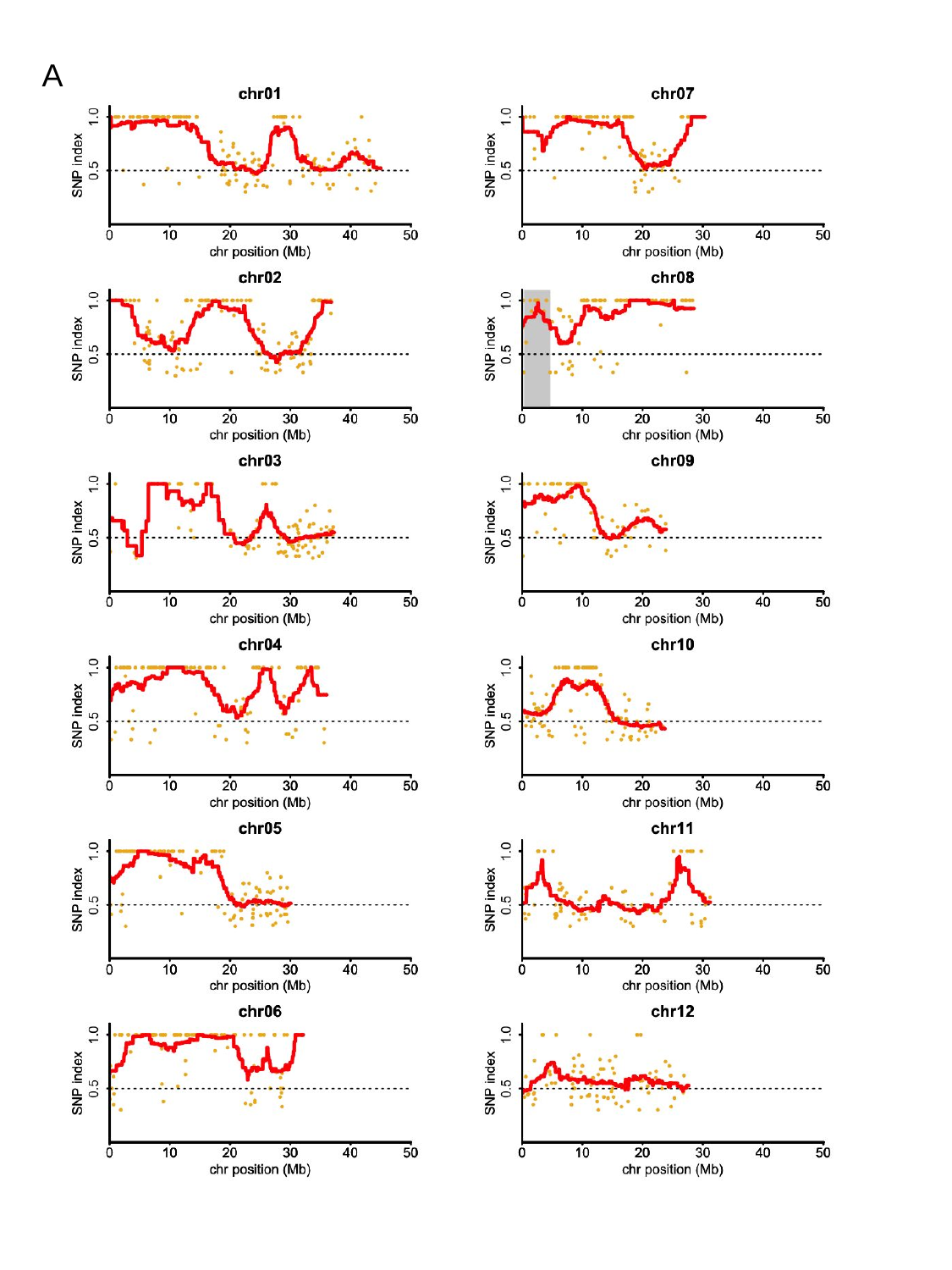

A

## Slide 2
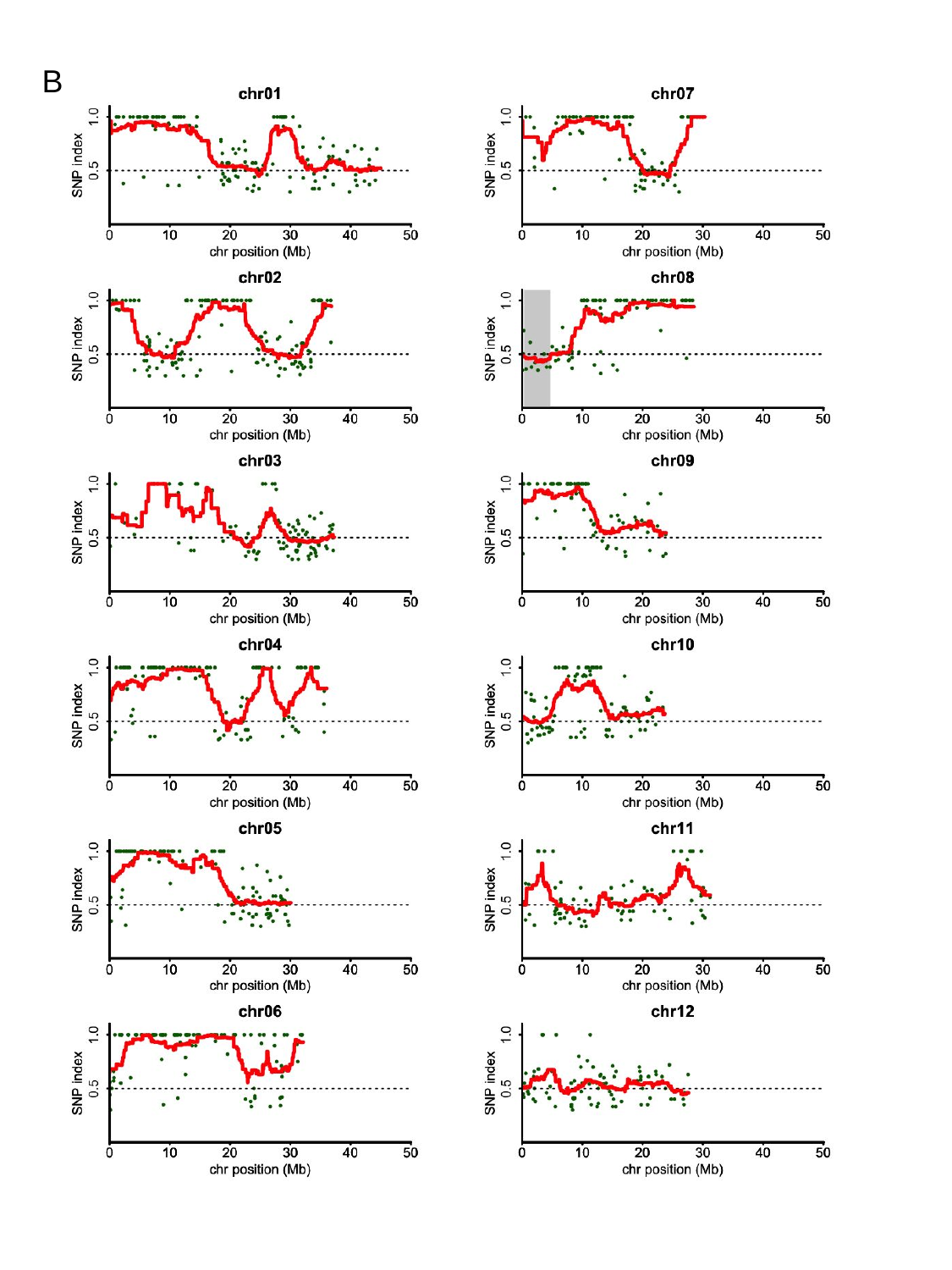

B

## Slide 3
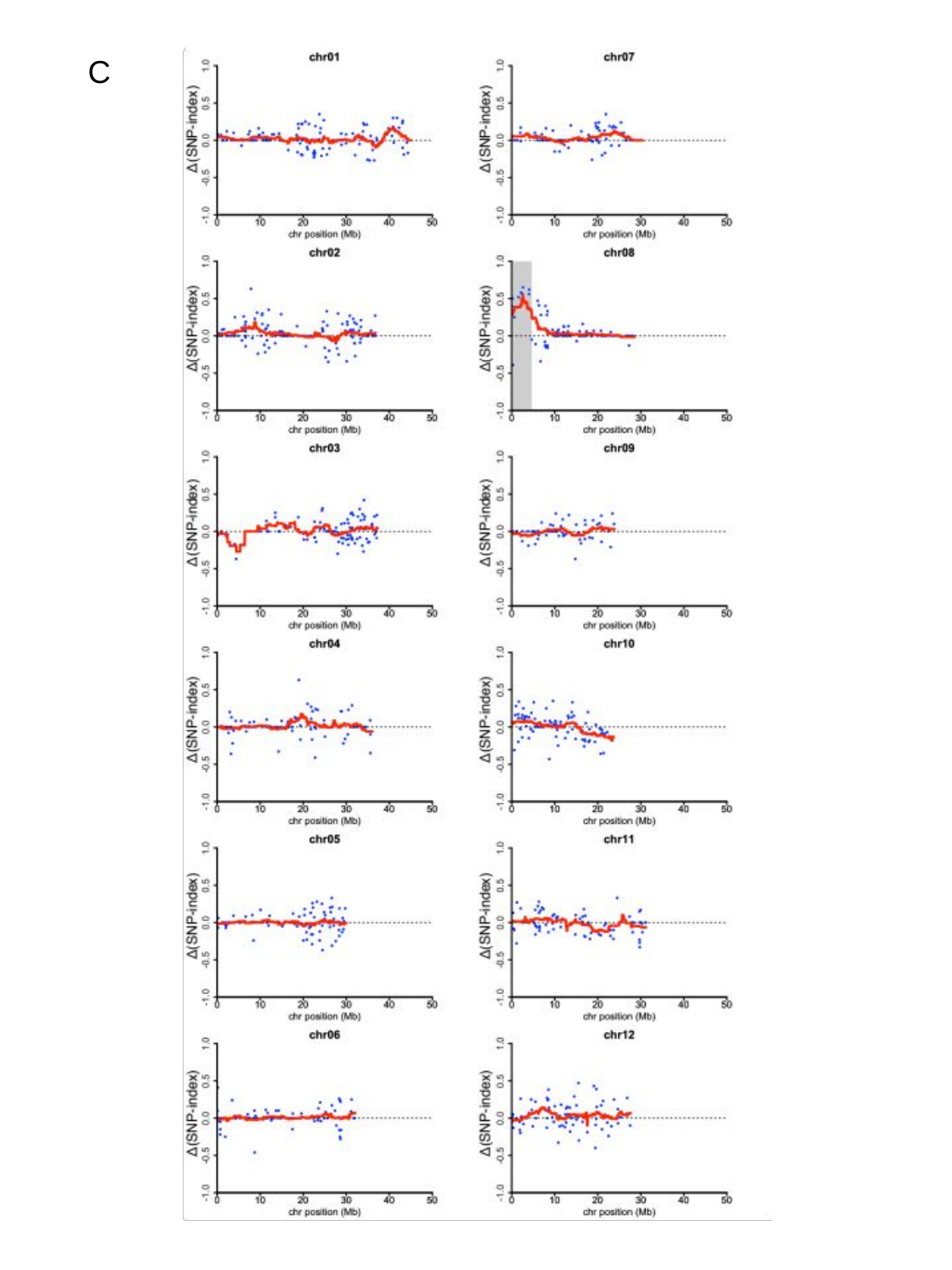

C

Supplement: Figure S4 — MutMap+ identifies the genomic region harboring the causative mutation of Hit11440. (A) SNP-index plot of mutant bulk, (B) SNP-index plot of wild-type bulk, and (C) Δ(SNP-index) plot obtained by subtraction of wild-type bulk SNP-index from mutant bulk SNP-index for all the 12 rice chromosomes. Red lines represent the sliding window average of 4 Mb interval with 10 Kb increment. Shaded areas correspond to the candidate genomic region where mutant and wild-type SNP-indices exhibit statistically significant (P < 0.05) differences (i.e. Δ(SNP-index) > 0). (PPTX) [file pone.0068529.s004.pptx]

## Slide 1
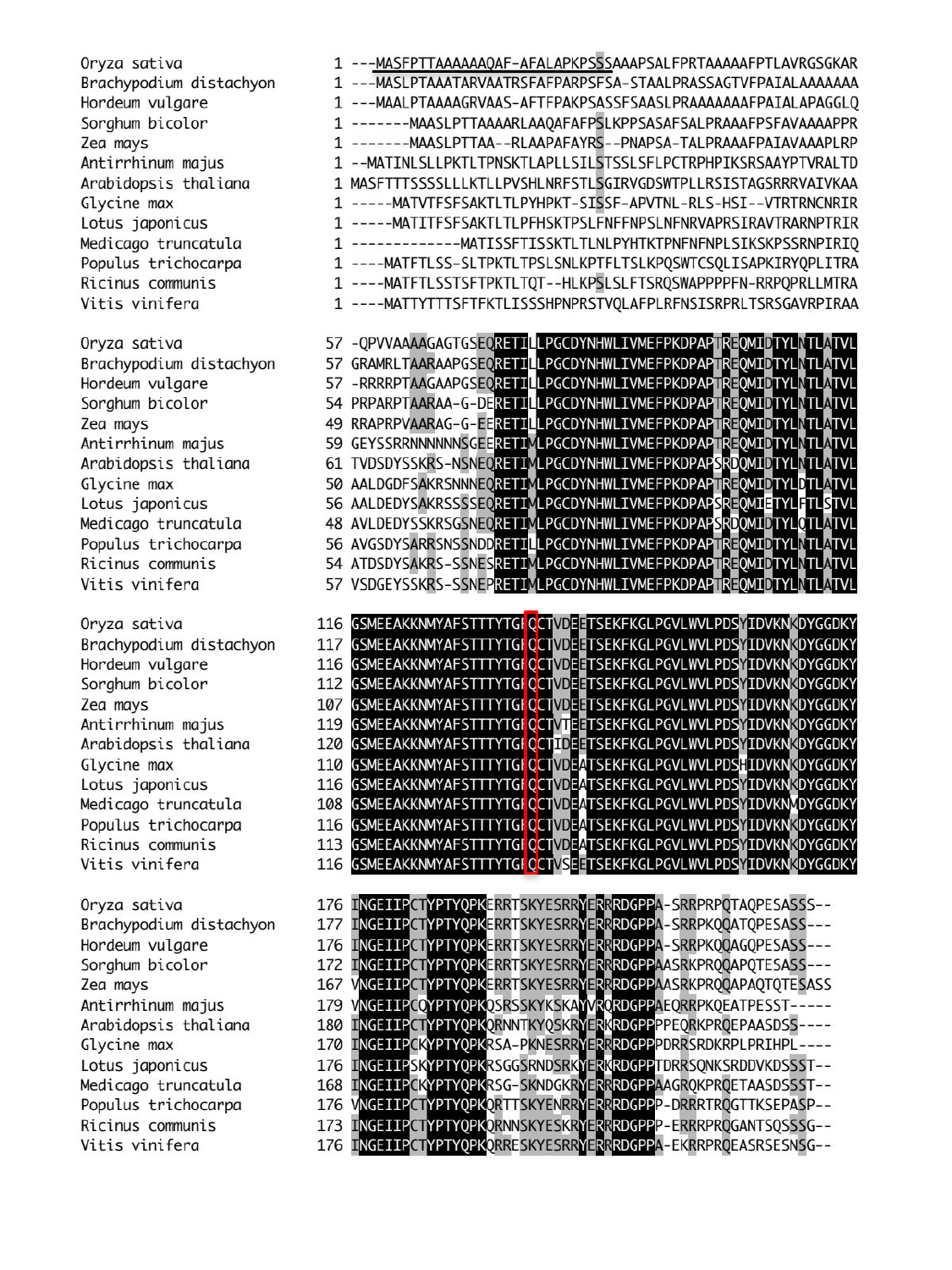

Supplement: Figure S5 — Alignment of DAG proteins from multiple plant species. The predicted 27-aa chloroplast residue in rice is underlined, and the highly conserved mutated glutamine (Q) residue in Hit11440 is indicated by a red box. NCBI reference numbers of the sequences used: Oryza sativa (NP_001060965.1), Brachypodium distachyon (XP_003573360.1), Hordeum vulgare (BAJ99034.1), Sorghum bicolor (XP_002445021.1), Zea mays (ACN27869.1), Antirrhinum majus (Q38732.1), Arabidopsis thaliana (AAM65001.1), Glycine max (ACU13265.1), Lotus japonicus (AFK45843.1), Medicago truncatula (XP_003593397.1), Populus trichocarpa (XP_002316698.1), Ricinus communis (XP_002518590.1), and Vitis vinifera (XP_002283211.1). (PPTX) [file pone.0068529.s005.pptx]
