# Supplementary material for: MutMap+: Genetic Mapping and Mutant Identification without Crossing in Rice
Source: PLoS One. 2013 Jul 10;8(7):e68529. doi: 10.1371/journal.pone.0068529 (PMC3707850; doi:10.1371/journal.pone.0068529)
Supplement: Figure S3 — Alignment of NAP6 homologs from multiple plant species. Identical and similar amino acids are indicated in black and gray backgrounds, respectively. Position of the mutated alanine residue in Hit9188 is given by a red box. (PPTX) [file pone.0068529.s003.pptx]

## Slide 1
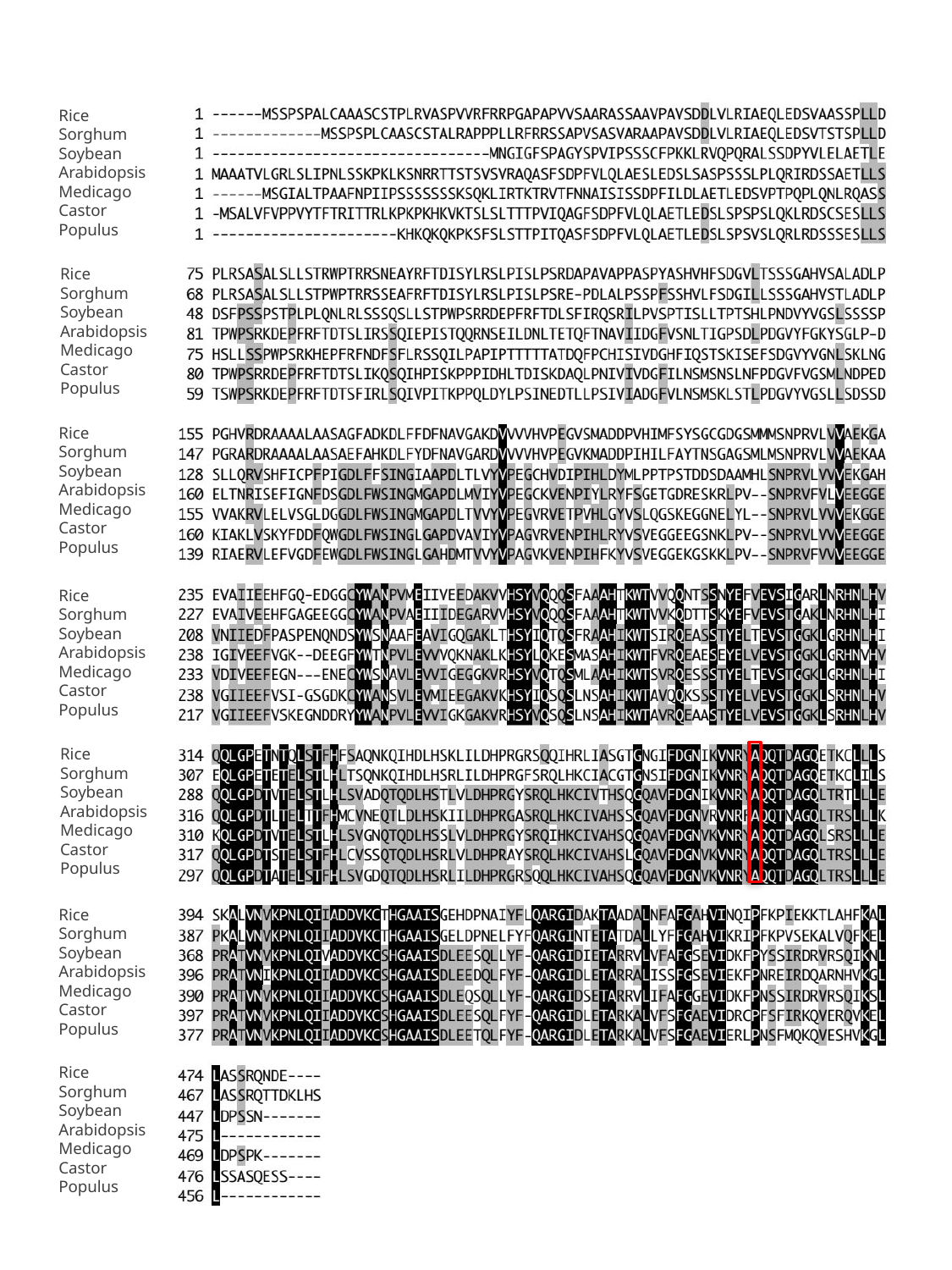

Rice
Sorghum
Soybean
Arabidopsis
Medicago
Castor
Populus
Rice
Sorghum
Soybean
Arabidopsis
Medicago
Castor
Populus
Rice
Sorghum
Soybean
Arabidopsis
Medicago
Castor
Populus
Rice
Sorghum
Soybean
Arabidopsis
Medicago
Castor
Populus
Rice
Sorghum
Soybean
Arabidopsis
Medicago
Castor
Populus
Rice
Sorghum
Soybean
Arabidopsis
Medicago
Castor
Populus
Rice
Sorghum
Soybean
Arabidopsis
Medicago
Castor
Populus
