# Supplementary material for: MutMap+: Genetic Mapping and Mutant Identification without Crossing in Rice
Source: PLoS One. 2013 Jul 10;8(7):e68529. doi: 10.1371/journal.pone.0068529 (PMC3707850; doi:10.1371/journal.pone.0068529)
Supplement: Table S1 — Summary of Illumina GAIIx whole genome sequencing results of Hit9188 and Hit11440 M3 mutant and wild-type bulks. (DOCX) [file pone.0068529.s006.docx]

**Table S1.**

Summary of Illumina GAIIx whole genome sequencing results of Hit9188 and Hit11440 M3 bulks.

| Mutant | Sample | Number M3 individuals bulked | Number of Illumina short reads | Total sequence (Gb) | Genome coverage (%)^a^ | Mean depth^b^ |
| --- | --- | --- | --- | --- | --- | --- |
| Hit9188 | Mutant bulk | 40 | 95,552,424 | 7.17 | 97.4 | 16.5 |
|  | Wild-type bulk | 40 | 57,925,150 | 4.34 | 95.2 | 9.9 |
| Hit11440 | Mutant bulk | 20 | 67,392,352 | 5.05 | 96.7 | 12 |
|  | Wild-type bulk | 20 | 62,529,426 | 4.68 | 97.8 | 11.4 |

Illumina short reads were aligned to Hitomebore consensus sequence as described in Material and Methods.

^a^Percentage of total genomic region of Hitomebore reference sequence aligned by short reads.

^b^Average read depth over the whole genome.
