# Supplementary material for: MutMap+: Genetic Mapping and Mutant Identification without Crossing in Rice
Source: PLoS One. 2013 Jul 10;8(7):e68529. doi: 10.1371/journal.pone.0068529 (PMC3707850; doi:10.1371/journal.pone.0068529)
Supplement: Table S2 — SNPs with SNP-index 1 within the candidate genomic region detected on chromosome 1 that exhibited statistically significant (Fisher’s exact test: P <0.05) differences between Hit9188 mutant and wild-type bulk sequences. (DOCX) [file pone.0068529.s007.docx]

**Table S2.**

SNPs with SNP-index of 1 within the candidate genomic region detected on chromosome 1 that exhibited statistically significant (Fisher's exact test: *P*<0.05) differences between Hit9188 mutant and wild-type bulk sequences.

| Chr. | Position | Reference base^a^ | Altered base | Depth | Mutated gene | Amino acid change |
| --- | --- | --- | --- | --- | --- | --- |
| 1 | 974469 | G | A | 6 | No hit | - |
| 1 | 1234738 | C | T | 26 | Os01g0121800 (Glycosyl transferase, family 14) | P to L |
| 1 | 1379378 | C | T | 5 | No hit | - |
| 1 | 1503571 | C | T | 18 | Os01g0127300 (SufBD family) | A to T |
| 1 | 1523129 | C | T | 27 | No hit | - |
| 1 | 1674496 | C | T | 20 | Os01g0130000 (Cation efflux protein family) | None (intron) |
| 1 | 3025549 | C | T | 6 | No hit | - |
| 1 | 3485142 | C | T | 5 | No hit | - |

^a^Hitomebore consensus sequence was used as a reference.
