# Supplementary material for: MutMap+: Genetic Mapping and Mutant Identification without Crossing in Rice
Source: PLoS One. 2013 Jul 10;8(7):e68529. doi: 10.1371/journal.pone.0068529 (PMC3707850; doi:10.1371/journal.pone.0068529)
Supplement: Table S3 — SNPs with SNP-index 1 within the candidate genomic region detected on chromosome 8 that exhibited statistically significant (Fisher’s exact test: P <0.05) differences between Hit11440 mutant- and wild-type bulk sequences. (DOCX) [file pone.0068529.s008.docx]

**Table S3.**

| Chr. | Position | Reference base^a^ | Altered base | Depth | Mutated gene | Amino acid change |
| --- | --- | --- | --- | --- | --- | --- |
| 8 | 1033215 | G | A | 11 | No hit | - |
| 8 | 1329152 | G | A | 12 | No hit | - |
| 8 | 1904318 | G | A | 6 | No hit | - |
| 8 | 2114429 | G | A | 13 | No hit | - |
| 8 | 2178176 | G | A | 15 | Os08g0139100 (Similar to DAG protein, chloroplast precursor) | Q to Stop |
| 8 | 2498012 | G | A | 18 | No hit | - |
| 8 | 2634520 | G | A | 17 | No hit | - |
| 8 | 2812184 | G | A | 8 | No hit | - |
| 8 | 4046217 | G | A | 11 | No hit | - |
| 8 | 4091705 | G | A | 20 | No hit | - |

SNPs with SNP-index 1 within the candidate genomic region detected on chromosome 8 that exhibited statistically significant (Fisher's exact test: *P*<0.05) differences between Hit11440 mutant and wild-type bulk sequences.

^a^Hitomebore consensus sequence was used as a reference.
